# Working conditions in hospitals revisited: A moderated-mediated model of job context and presenteeism

**DOI:** 10.1371/journal.pone.0205973

**Published:** 2018-10-22

**Authors:** Merce Mach, Aristides I. Ferreira, Luis F. Martinez, Antonina Lisowskaia, Grace K. Dagher, Amalia R. Perez-Nebra

**Affiliations:** 1 Department of Business, Faculty of Economics and Business, University of Barcelona, Barcelona, Spain; 2 Department of Human Resources and Organizational Behaviour, ISCTE-IUL, Instituto Universitário de Lisboa, Lisboa, Portugal; 3 Department of Management, Nova School of Business and Economics, Universidade Nova de Lisboa, Lisboa, Portugal; 4 Organizational Behavior and Personnel Management Department, Graduate School of Management, St. Petersburg State University, St. Petersburg, Russia; 5 Department of Management Studies, Adnan Kassar School of Business, Beirut Campus, Lebanese American University, Beirut, Lebanon; 6 Department of Psychology, Centro Universitario de Brasilia, Campus Asa Norte, Brasilia, Brazil; Massachusetts Department of Public Health, UNITED STATES

## Abstract

This study examines whether the relationship between the employees’ perceived job autonomy may be prone to the contextual influence of supervisor support and presenteeism climate in explaining the attendance behaviors of presenteeism–the employees’ decision to attend work despite being ill or not feeling well. Does work context play a role on presenteeism climate and the specific act of presenteeism? This study includes 213 health care employees (e.g., nurses, doctors) working in one private hospital in Lebanon. We used the ordinary least squared (OLS) regressions path analytical framework and bootstrapping methods to estimate the hypothesized moderated-mediation models. Our findings indicate that healthcare job resources (job autonomy) is correlated with the presenteeism climate and the occurrence of presenteeism attendance behaviors. We also found that this relationship is mediated by presenteeism climate and that supervisor support moderates the observed indirect relationship. This study extends the organizational attendance research domain to presenteeism climate by explaining for both doctors and nurses how contextual variables explains the relationship between jobs resources and presenteeism attendance behaviors. Supervisor support plays an important role in encouraging task autonomy and thus allowing employees increase their perception of empowerment to manage their actions at work. Overall, healthcare managers should ensure that employees understand their roles and duties and have an up-to-date, clearly defined role (e.g., job description) so that they can meet their organizations’ goals.

## Introduction

The job demands-resources model has been extensively studied in management literature [1], integrating important variables and organizational outcomes such as job engagement and burnout. Despite the well-known relationship between burnout and presenteeism [2], as well as between the job demands-resources model and burnout [3], [4], to the best of the authors’ knowledge, no studies have attempted to integrate presenteeism as a comprehensive outcome of this model, taking into account the healthcare sector context and its specificities. The purpose of this study is to assess attendance dynamics (i.e., presenteeism at work) as a function of work characteristics, individual differences and organizational contexts. Thus, we aim to examine the influence of job resources on presenteeism.

Johns [5] presented a comprehensive review on presenteeism and proposed a model which features the work context, specific employee characteristics and some aspects of the work experience. Presenteeism also has important consequences on organizational performance and individuals’ wellbeing and is more prevalent in the healthcare and educational sectors [2], [6], [7]. Bergström and colleagues [8] showed that employees who reported attending work frequently while sick were, in fact, significantly more absent in the following 18 months and even three years later. Presenteeism can also damage the quality of the service provided. In this respect, presenteeism among physicians may affect the quality of healthcare provided [9], [10], the quality of the work itself [11], and the long-term health of the incumbents [8].

Although the literature has covered the hidden costs of presenteeism [12], [13] and its occurrence among certain groups of people[6], there are fewer explanations regarding why presenteeism occurs and whether people who go to work while ill may experience contextual pressure (e.g., a normative cultural climate favoring presenteeism) or self-serving motives (e.g., no backup for their expertise).

By linking job resources (job autonomy), and supervisor support to presenteeism attendance behavior, this study seeks to respond to the call to further examine the role that presenteeism climate plays [14] and test the effect of manager support on the influence of contextual variables on presenteeism attendance behavior. In line with previous studies reinforcing the moderator role of supervisor support [15], this study tests: (a) whether supervisor support moderates the mediation of presenteeism climate in the relationship between contextual variables and presenteeism attendance behaviors and; (b) how the social context (presenteeism climate) influences the relationship between job resources (autonomy) and the occurrence of presenteeism behavior.

### Presenteeism and the work context

Work context refers to the physical and social factors that influence the nature of work [16], such as job resources [5], [17]. Recent research has acknowledged an increase in the average level of work intensity, determining that almost half of current jobs are potentially unhealthy places to work due to their poor intrinsic quality, or their working-time quality [18]. This forces employees to be present at work even if suffering from physical or psychological conditions. The sense of obligation to attend work regardless of their medical conditions depends on the context in which this attendance episode occurs [19], [20].

Different factors may exert an influence on the employees’ decision to go to work while ill. Employees might have good intentions but they are driven by duty, and both the significance and the importance of their jobs [21], or quite simply, they care about not letting colleagues down. Also, employees might go to work regardless of not feeling well or suffering from physical or psychological health conditions. Morgeson and Humphrey [22] presented a research call exploring work configurations that extend the influence of the job context (task and social factors), as these might influence employees’ attendance decisions.

Lu and colleagues [23] summarized recent organizational studies and pointed out that features in the work contexts (e.g., easy job replacement) and personality traits (e.g., neuroticism and the internal locus of control) are related to the act of presenteeism [20], [23], [24]. They also indicate that these factors played a potential role as buffers in mitigating or exacerbating the impact of presenteeism on employees [23]. However, no studies analyze the influence of presenteeism climate and the potential intervening effect of supervisor support on the relationship between job resources on the attendance at work.

The job demands-resources model describes how employee wellbeing is influenced by work environment characteristics [3]. Scholars have used it to elucidate the benefits and disadvantages of work contexts, suggesting that strain is a response to imbalance between the demands and resources an employee has to deal with [17]. Accordingly, job demands may lead to impaired health because they can weaken employees’ personal resources (mental or physical), thus leading to health problems [1] [4].

Job resources may trigger motivational processes which can mitigate the impact of job demands [25]. They can also help employees cope with work demands and job ambiguity [3], [4]. Job resources are “those physical, psychological, social, or organizational aspects of the job that may reduce job demands and the associated physiological and psychological costs; [they] can be functional in achieving work goals, and stimulate personal growth, learning and development” (p. 501) [26]. Thus, job characteristics allow employees to reduce the costs associated to job demands and all the obstacles to achieve their goals, learning and performance at work [17]. Moreover, job demands and job resources are negatively associated: specifically, high levels of job resources–such as supervisor support–tend to reduce task ambiguity [1], [27]. Thus, receiving support from supervisors is vital for healthcare employees [28], [29].

### The healthcare sector in Lebanon

In this study, we focus on the healthcare sector in Lebanon. The private sector currently provides 90% of healthcare services in the country. Consequently, the overall annual expenditure per person is $499, thus in line with the expenditure in Europe and North America–a very high price tag. However, the health indicators in Lebanon are in line with other countries in the region. Specifically, life expectancy for men is 69 and 72 for women, while the infant mortality rate is 27 per 1000 births [30], [31].

Healthcare in the private sector is provided through hospitals, clinics, private laboratories and NGO-owned public healthcare centers. Since the health sector is growing in an unregulated milieu, investments are uncontrolled and suppliers induce demand. Consequently, there is an oversupply of beds, high-tech equipment and specialized doctors, but an undersupply of nurses due to the job’s poorly perceived professional status. Moreover, private hospitals do not offer the same quality of services to the poor as they provide their wealthy clients. Occasionally, private hospitals collect extra fees from patients that are admitted through a contract with the Ministry of Health or other public funds [30], [31], [32].

Healthcare in the (residual) public sector is provided by public primary healthcare centers and dispensaries, in addition to non-individual preventive care through health education and screening campaigns. Public hospitals are overstaffed and operate under rigid administrations. Furthermore, they cannot compete in the market. The government passed a Public Hospitals Autonomy Law in 1996 which seems to have led to an increase in admissions and patient satisfaction [30], [31], [32].

Taking into account that professionals in the Lebanese healthcare sector have to deal with an occasional lack of resources [32], we opted to include the job resource branch of the job demands-resource model in our study. In order to deal with political constraints, hospital managers are more prone to develop climates of presenteeism, where pressure to go to work despite being ill is very encouraged [14], [33]. As political and financial constraints are culturally prominent in Lebanon, we aim to evaluate the presenteeism culture among a sample of healthcare professionals.

When employees in the healthcare sector perceive a lack of job resources, they will find it more difficult to achieve the desirable outcomes [25]. This promotes a sense of frustration and leads to a lack of motivation, low levels of commitment, and withdrawal from work [3]. Nevertheless, job demands connected to social support might also predict extra-role performance [34]. Therefore, the literature shows that Lebanon scores high on Power Distance (score of 75), meaning that employees work in very hierarchical structures and tend to accept power inequalities and centralization [34]. Thus, resources such as autonomy help employees to deal with presenteeism climates, which in turn affect their job attendance behaviors. As there is previous evidence regarding the moderator role of supervision support on presenteeism outcomes [23], [27], in this study we will focus on the moderator role of supervisor support on the indirect relationship between job autonomy and presenteeism attendance through the mediation of presenteeism climate. In sum, the job demands-resource model [17] provides the rationale for this study, with the job resources construct as both “job autonomy” and “supervisor support” explaining the indirect relationship with presenteeism attendance. Next, we present our research hypotheses:

***H1***: *Presenteeism climate mediates the relationship between job resources (work autonomy) and the number of days employees go to work while ill*.
***H2*:** *The strength of the mediated relationship between job resources (job autonomy) and the number of days employees go to work ill (due to presenteeism climate) will vary depending on the existing level of supervisor support*. *As such*, *the direct and the indirect effect of job resources via presenteeism climate in predicting the number of days present though ill will be stronger with high levels of supervisor support*.

The literature shows that the healthcare sector hierarchy is highly dominated by doctors and organizations are characterized by a bureaucratic management style [35]. In this sense the nursing profession in power distance cultures as Lebanese is highly conditioned upon the support received from their supervisors [35] and the level of autonomy perceived. Also, job autonomy plays an important role contributing to nurses’ work engagement [15]. As work engagement is correlated with presenteeism attendance [34] we hypothesized that the effects observed for the general sample is more prevalent with professionals who have less access to decision-making roles (e.g., nurses), and therefore, their sickness presence is more dependent on their level of autonomy and supervisor support.

**Fig 1:**
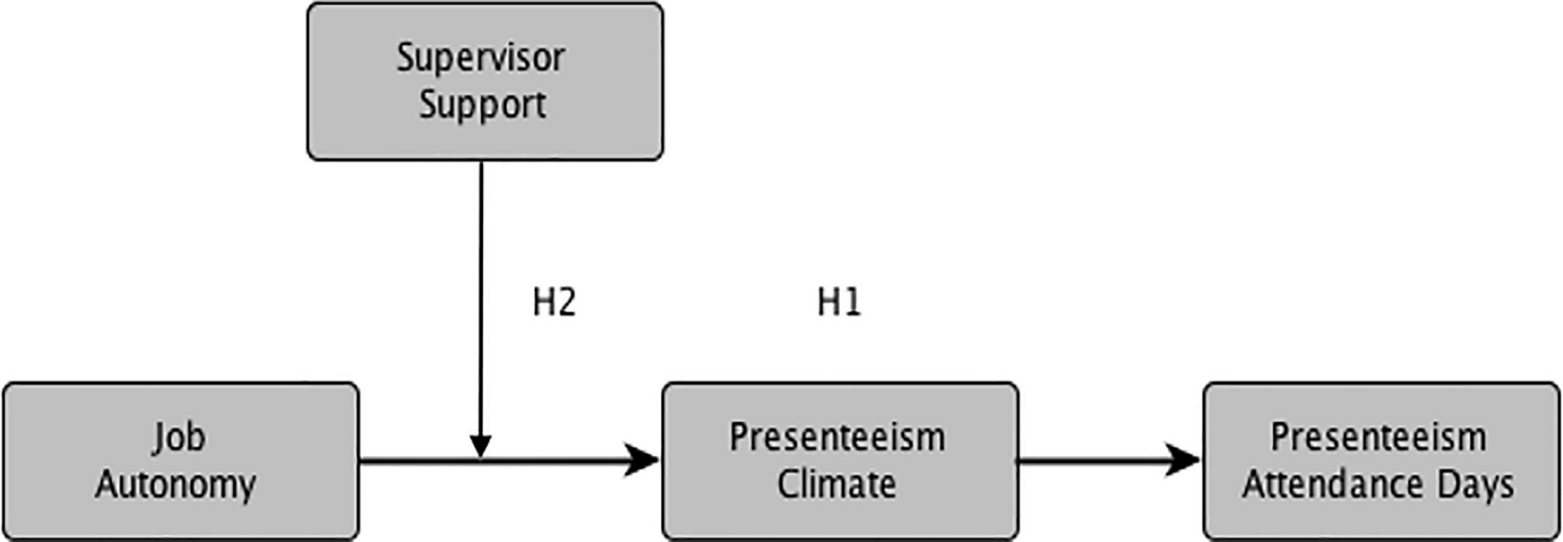
The moderated–mediation model of job context and presenteeism attendance behavior.

Fig 1 depicts the hypothesized model and hypotheses.

## Method

### Sample and data collection

Study participants included employees from the healthcare sector (nurses, doctors, and staff), working at a large private hospital in the Lebanon. Of the study participants, 38% were male. The mean age of participants was 33.08 (*SD* = 8.1). The average seniority (years of experience) was 7.82 years (*SD* = 7.4).

We asked permission from the hospital’s human resource department to distribute 350 questionnaires. Participation was voluntary and confidential. We asked participants to give consent to participate through the cover letter. Previously, the survey was approved by the University ethics committee in accordance with the NCSR (National Council for Scientific Research) in Lebanon. 213 healthcare professionals filled out a questionnaire (response rate of 60.85%) during the second half of year 2013. Constructs were appraised based on existing previous validated scales and the English was the language of the questionnaire.

### Measures

Respondents indicated their agreement with each statement using a seven-point Likert-scale ranging from 1 (strongly disagree) to 7 (strongly agree).

#### Organizational presenteeism climate

The climate of presenteeism was assessed through 9 items from the presenteeism culture questionnaire developed by Ferreira and colleagues [14]. Higher scores represent a higher level of organizational presenteeism climate. Cronbach’s alpha is 0.81. Sample item included: *“When suffering from health problems*, *I think that I should request permission to be absent from work*, *but I choose to attend my job”*

#### Job autonomy

To measure the perception of job autonomy, we used 3 items from the Job Diagnostic Survey subscale developed by Hackman, and Oldham [36]. Cronbach’s alpha is 0.90. A sample item was: “*I have considerable opportunity for independence and freedom in how I do my job*”.

#### Supervisor support

The level of perceived supervisor support was measured through 6 items from the scale developed by Oldham and Cummings [37]. Cronbach’s alpha is 0.92. Sample statement included: “*My supervisor helps me solve work-related problems*.*”*

#### Presenteeism days

Based on previous research [20], the presenteeism behavior dependent variable was measured by a single self-reported question: *“How many days did you go to work in the past six months even though you were sick or not feeling well*?*”*. We employed a fill-in-the-blank response format for the answers. Results were squared root.

#### Controls

Participants also provided some background information: their gender (where 1 was for male, and 2 for female), their age, their occupation or exact job title, the management position (where 1 was physician, 2 nurse, and 3 other staff), and their personal health status (using a Likert scale from 1“Poor health” to 5-“Excellent health”).

### Procedures

Before testing the hypotheses, the authors conducted preliminary analyses to check the measures’ psychometric properties and their discriminant validity. Then, the hypotheses were tested with *OLS* Ordinary Least Square through the *PROCESS* macro for SPSS, which assesses the conditional indirect effects [38]. *PROCESS* is a computational tool for path analyses-based moderations and mediation analyses as well as their combination as a “conditional process model”. In addition to estimating the model’s coefficients using an ordinary least squared (*OLS*) regression path analytical framework, *PROCESS* generates direct and indirect effects in mediation models and conditional indirect effects in moderated-mediation models [38]. Thus, this conditional process modeling is the integration of moderation and mediation analyses into a unified analytical model. This macro also facilitates the recommended bootstrapping methods–a random sampling with replacement which allows assigning measures of accuracy (e.g. confidence intervals) to sample estimates–and it also provides a means to probe the significance of the conditional indirect effect.

### Measurement model validity

Next, the authors conducted a parallel analyses based on minimum rank factor analysis[39] and confirmed the structure suggesting the three hypothesized constructs of presenteeism climate, job autonomy and supervisor support. Moreover, the data reinforced that a three-factor structure (with residuals covariance after checking modification indices > 8.0) offered a better goodness of fit statistically and distribution of residuals [*χ2*(153) = 465.079, *p* < 0.01, *CFI* = 0.91, *IFI* = 0.91, *RMSEA* = 0.09] than alternative models, with all items loading in a single factor [*χ2*(160) = 1433.298, *p* < 0.01, *CFI* = 0.62, *IFI* = 0.62, *RMSEA* = 0.19].

Data regarding the measurement model’s validity, shows that composite reliability scores were equal or higher than 0.70 (0.86–0.93) for the three studied variables [40]. There is support for convergent validity, with all the average variance extracted (*AVE*) being higher than 0.50 (0.68–0.82). This suggests a strong correlation with other items within the same hypothetical construct. Additionally, convergent validity was reinforced, with all the values higher than the maximum shared variance (*MSV*). The data also confirmed discriminant validity, with all the average shared variance (*ASV*) scores below the *AVE* score [40].

## Results

Table 1 includes descriptive statistics, zero-order correlation, and reliability coefficients.

**Table 1:** Descriptive statistics and correlations.

	*Mean*	*SD*	*1*	*2*	*3*	*4*	*5*	*6*	7
1. Gender	1.62	0.49	—						
2. Age	33.1	8.02	-0.11	—					
3. Supervisor (*position*)	1.75	0.44	0.19^**^	-0.43^**^	—				
4. Health status	4.60	0.60	0.14^*^	-0.41^**^	0.23^**^	—			
5. Job Autonomy	5.92	0.69	-0.08	0.03	-0.04	0.04	(0.85)		
6. Presenteeism Climate	4.75	0.90	0.06	-0.03	0.02	-0.01	0.21^**^	(0.83)	
7. Supervisor Support	5.52	1.03	0.06	0.02	-0.08	0.07	0.35^**^	0.49^**^	(0.94)
8. Presenteeism days *(last 6 month)*	4.50	0.50	0.08	0.08	-0.03	-0.26^**^	0.14^*^	0.18^**^	**0.11**^†^

*Note*. N = 213.

Significant at

**p< 0.01

*p< 0.05

^†^*p<* 0.1

Cronbach’s alpha is shown in the diagonal.

### The indirect effect of job context through presenteeism climate

Hypothesis 1 predicted the mediation role of presenteeism climate on the relationship between job autonomy and the occurrence of presenteeism behavior. Carrying out a simple mediation analysis of the overall hospital sample, findings provide evidence that job autonomy indirectly influenced presenteeism behavior through its effects on presenteeism climate. Table 2 shows the influence that job autonomy has on presenteeism climate (*a =* 0.32) and that of presenteeism climate on presenteeism behavior (*b =* 0.09). A bias-corrected bootstrapping 95% confidence interval (*CI*) for the indirect effect (*ab =* 0.03) based on 10,000 bootstrap samples was entirely above zero [0.01 to 0.07]. Moreover, there is no evidence that job autonomy influenced presenteeism behavior independently of its effect on presenteeism climate (*c’* = 0.09; *p =* 0.09), thus, supporting Hypothesis 1.

**Table 2:** Model coefficients for the mediation models of presenteeism climate ^a^.

**Hospital (*n* = 213)**	***Consequent*** ^***b***^						
	***M* (*Presenteeism climate*)**				***Y (Presenteeism days)***		
***Antecedents*** ^***b***^:	***Coeff*** ^***b***^	***SE***	***p***		***Coeff*** ^***b***^	***SE***	***p***
***X (Job autonomy)***	0.32**	0.10	.00		**0.09**^**†**^	0.05	.09
***M (Presenteeism climate)***	—	—	—		0.09**	0.04	.01
***Constant***	2.36*	1.02	.05		1.35**	0.50	.01
	*R*^*2*^ *=* 0.068				*R*^*2*^ *= 0*.*136*		
	*F* (4, 178) = 3.225**, *p<* .*01*				*F* (5, 177) = 5.593***, *p<* .*001*		
	***Total & Direct effects***				***Indirect effect of X on Y***		
	***Effect***	***SE***	***t***	***p***	***Effect***	***Boot SE***	***Bias corrected &*** ***accelerated CI***
**Total effect of X on Y**	0.12*	0.05	2.37	.02	**0.03**	**0.02**	**[.01, .07 ]**
**Direct effect of X on Y**	**0.09**^**†**^	0.05	1.72	.09			
**Nurses (*n* = 85)**	***Consequent*** ^***b***^						
	***M* (*Presenteeism climate*)**				***Y* (*Presenteeism days*)**		
***Antecedents*** ^***b***^:	***Coeff***^** **^^***b***^	***SE***	***p***		***Coeff*** ^***b***^	***SE***	***p***
***X (Job Autonomy)***	0.34*	0.16	.04		0.19	0.06	ns
***M*** *(****Presenteeism climate****)*	—	—	—		0.11*	0.04	.02
***Constant***	1.13	2.0	ns		1.59*	0.76	.04
	*R*^*2*^ *=* 0.070				*R*^*2*^ *=* 0.223		
	*F* (4, 80) = 1.502, *p* = ns				*F* (5, 79) = 4.546***, *p<* .*001*		
	***Total & Direct effects***				***Indirect effect of X on Y***		
	***Effect***	***SE***	***t***	***p***	***Effect***	***Boot SE***	***Bias corrected & accelerated CI***
**Total effect of X on Y**	0.55	0.06	0.88	ns	**0.04**	**0.03**	**[.01, .11 ]**
**Direct effect of X on Y**	0.02	0.06	0.30	ns			
**Staff (*n* = 72)**	***Consequent*** ^***b***^						
	***M* (*Presenteeism climate*)**				***Y (Presenteeism days)***		
***Antecedents*** ^***b***^:	***Coeff*** ^***b***^	***SE***	***p***		***Coeff*** ^***b***^	***SE***	***p***
***X (Job autonomy****)*	0.54*	0.21	.02		-0.08	0.11	ns
***M*** *(****Presenteeism climate****)*	—	—	—		**0.14**^**†**^	0.07	.07
***Constant***	**2.72**^**†**^	1.47	.07		-0.19	0.78	ns
	*R*^*2*^ *=* 0.136				*R*^*2*^ *= 0*.*199*		
	*F* (4, 50) = 1.975, *p = ns*				*F* (5, 49) = 2.433*, *p<* .*05*		
	***Total & Direct effects***				***Indirect effect of X on Y***		
	***Effect***	***SE***	***t***	***p***	***Effect***	***Boot SE***	***Bias corrected &*** ***accelerated CI***
**Total effect of X on Y**	- 0.01	0.11	**-**0.02	ns	**0.07**	**0.05**	**[.01, .21 ]**
**Direct effect of X on Y**	- 0.07	0.11	-0.66	ns			

^**a**^ Significant at:

*** p< .001

** p< .01

* p< .05

^†^ *p<* .1

^b^ *Coeff* = Regression coefficients; *SE* = Standard error; *CI* = Confidence interval; *X* = Antecedent variable; *M* = Mediator; *Y* = Dependent variable. Control variables included as covariates were gender, age, supervisor position and health status. (*CIs* containing zero are interpreted as non-significant).

Testing this hypothesis by the different subsamples (e.g., nurses, physicians and staff), we corroborate findings for nurses and staff subgroups of respondents. However, due to the small sample size of physicians (*n* = 35); we do not found significant relationships. Results detailed in Table 2 show that job autonomy correlates with presenteeism climate (*a =* 0.34 for nurses sample; *a =* 0.54 for staff sample) and that presenteeism climate relates to presenteeism behavior (*b =* 0.11 for nurses sample; *b =* 0.14 for staff sample). The 95% CI [0.01, 0.11] for the indirect effect of nurses (ab = 0.034) was entirely above zero, as well as for staff sample; the 95% CI [0.01, 0.21] for the indirect effect of staff (ab = 0.07). These results support partially hypothesis 1 when testing by subsamples.

### The moderating effect of supervisor support

Hypotheses H2 predicted that the indirect effect (i.e., mediated by presenteeism climate) of job autonomy on presenteeism attendance days would vary as a function of perceived supervisor support. Accordingly, we performed moderated mediation regression analyses [Model 8] [38].

As shown in Table 3, the indirect effect of job autonomy on presenteeism attendance days (H2) via presenteeism climate was significant only for employees with high (+1*SD*) or low (-1*SD*) supervisor support. In both cases, the 95% *CI* [0.01, 0.08] and [-0.06, -0.01] for the conditional indirect effect did not contain zero. These findings show that job autonomy interacts with supervisor support to explain the variance of the presenteeism climate, which, in turn, relates to presenteeism attendance days. Thus, hypothesis 2 is supported.

**Table 3:** Conditional direct & indirect effects of job autonomy on presenteeism behaviors ^a^.

***Antecedent***	***Consequent***			
***X (Job autonomy)***	***M* (*Presenteeism Climate*)**		***Y* (*Presenteeism Days*)**	
	*R*^*2*^ *=* 0.339		*R*^*2*^ *=* 0.139	
	*F*(6, 176) = 15.06***, *p<* .*001*		*F*(7, 175) = 4.042***, *p<* .*001*	
**Conditional** **DIRECT** **effect of *X (Job autonomy)* on *Y (Presenteeism days)*** **at values of the moderator (*W*)**				
***Supervisor*** ***Support (W)*** ^*b*^	***Effect***	***SE***	***t***	***p***
-1SD	0.05	0.07	0.71	ns
Mean	0.09	0.05	1.62	ns
+1SD	0.12	0.09	1.42	ns
**Conditional** **INDIRECT** **effect of X on Y at values of the moderator** ^**a**^ **(W)**				
***Mediator (M)*:**	***Supervision*** ***Support (W)*** ^*b*^	***Effect***	***Boot SE***	***Bias corrected & accelerated CI***
***Presenteeism climate***	**-1SD**	**-0.02**	**0.01**	**[-.06, -.01 ]**
***Presenteeism climate***	Mean	0.01	0.01	[-.01, .05 ]
***Presenteeism climate***	**+1SD**	**0.05**	**0.03**	**[.01, .13 ]**

^a^ N *=* 213.

Significant at:

***p< .001

*X =* Antecedent variable; *M =* Mediator; *W* = Moderator; *Y =* Dependent variable. *SE =* Standard error; *CI* = Confidence interval. Control variables included as Covariates were gender, age, and health status.

^**b**^ Values for quantitative moderators (*W*) are the mean and plus / minus one SD from the mean.

Furthermore, when breaking into subgroups the study sample, the conditional indirect effect of job autonomy was significant only for nurses (Table 4). The contextual influence of supervisor support plays a significant role in explaining the indirect effect between job autonomy and presenteeism attendance days, through the role of presenteeism climate.

**Table 4:** Indirect effect of highest order product of job autonomy on presenteeism behaviors.

*Index of Moderated Mediation* [41]				
	*Mediator*	*Effect*	*SE* ^a^ *(Boot)*	*Bias corrected & accelerated CI*
**Hospital** (*n* = 213)	***Presenteeism climate***	0.035	0.20	**[.003, .083 ]**
**Nurses** (*n* = 85)	***Presenteeism climate***	0.036	0.25	**[.002, .099 ]**
**Physicians** (*n* = 38)	***Presenteeism climate***	0.043	0.69	[- .039, .270 ]
**Staff** (*n* = 72)	***Presenteeism climate***	0.056	0.60	[- .035, .201 ]

^**a**^ *SE =* Standard error

*CI* = Confidence interval. (*CIs* containing zero are interpreted as non-significant). Control variables included as covariates were gender, age, and health status.

**Fig 2:**
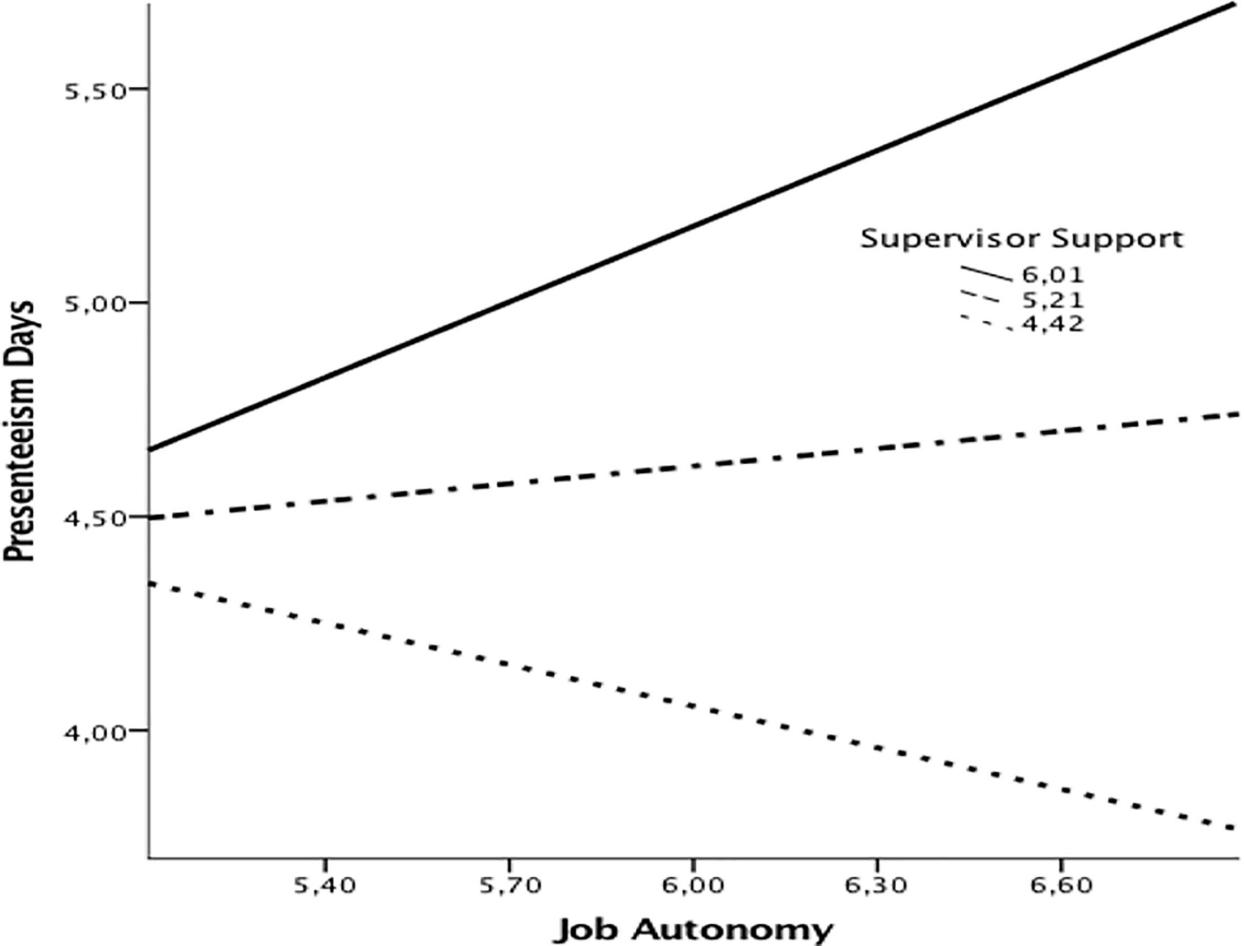
Interactive effect of job context and supervisor support on presenteeism attendance days through presenteeism climate.

The bootstrapping procedure results show that when supervisor support is not lacking, presenteeism climate mediates the relationship between job autonomy and presenteeism attendance days. This supports the theoretical argument that job autonomy interacts with supervisor support to influence presenteeism climate, which, in turn, impacts presenteeism attendance days. Hypothesis 2, therefore, is supported. Fig 2 shows the slopes representing the conditional indirect effect of job autonomy on presenteeism attendance days at values of the moderator–the supervisor support.

## Discussion

This study aimed to test whether the predictive role of job characteristics on employee attendance behaviors might be prone to contextual influences. The findings corroborate that perceived supervisor support does in fact moderate the relationship between perceived job resources (work autonomy) and attendance behaviours. In support of the hypotheses, the type of moderate mediation revealed that job autonomy was positively related to presenteeism behavior through presenteeism climate only for employees reporting about high or low levels of perceived supervisor support.

### Theoretical implications

In line with previous calls to extend research on the job demands-resources model and the attendance domain [14], [42], this study’s contribution is twofold. First, it expands the outputs of the job demands-resources model and the presenteeism climate domain to examine employees’ attendance behavior. Second, it emphasizes the importance of supervisor support. Without the perceived support from direct managers, the interplay between job demands and job resources cannot be buffered.

These results are in line with previous findings alerting to the fact that nurses believe that social support from their colleagues and supervisors might reduce the influence of challenging situations in the workplace [42], [43]. This is vital in the highly competitive environments that characterize the healthcare sector, where employees with low autonomy and role ambiguity perceive a lack of social support and knowledge sharing [44]. With the introduction of the mediator role of presenteeism climate in the current study, we provide a contribution to the presenteeism literature by empirically reinforcing the importance of studying the policies of presence (and the pressure to be present) in the healthcare sector [33].

Moreover, the findings also reveal the importance of increasing job autonomy in the healthcare sector where nurses and physicians have to deal with highly demanding contexts with limited power decision [35] and some degree of improvisation [45]. In line with previous studies [15] our findings reinforce the importance to develop job autonomy and social support, in order to promote positive work outcomes among nurses. Job autonomy plays an important role in poor health conditions [46] and contributes to the positive self-perception of wellbeing at work [47]. The findings were consistent with previous research that showed that job autonomy has a significant relationship with positive attitudes and job performance [48], and mental health [49], enabling healthcare professionals to deal with unexpected and stressful situations.

This study also contributes to the management literature by expanding the outputs of the job resources variables [3], [4] and showing how contextual work conditions explain the added variance in the occurrence of presenteeism attendance behaviors [14], [34]. Moreover, the study provides evidence of how several work context characteristics influence employee decisions to go to work while ill by analyzing the potential interplay of presenteeism organizational climate and supervisor support. These findings may also contribute to further develop theories within the field of wellbeing at work.

### Practical implications

The results from this study corroborate the effect work context has on the occurrence of presenteeism behavior and the explanatory moderated effect of supervisor support on this relationship [34]. Thus, healthcare organizations should create a working environment to decrease presenteeism among their employees (e.g., Active Rest Programmes [50] or Aerobic Physical Activity [51]) and preclude environments with climates where employees perceive low supervisor support, as they are more prone to adopt presenteeism attendance behaviors. In fact, supervisor support plays an important role in promoting task autonomy and thus allowing employees increase their perception of empowerment to manage their actions at work [1], [47]. Accordingly, managers should ensure that employees understand their duties and have an up-to-date, clearly defined job description, so that they can meet their organizations’ goals [11]. Other studies reinforce the importance to promote job autonomy to reduce burnout in the healthcare sector [52]. Therefore, healthcare managers should provide empowerment activities to less autonomous professionals such as nurses (e.g., each nurse could be responsible for several patients instead of specific activities). In other words, job description should be more flexible and encourage job crafting and the possibility to have more flexi-time schedules and policies.

### Limitations and future research

These findings are not without limitations. The convenience data used for the current study was collected by means of a self-reported questionnaire. Also, other measures of presenteeism should be considered in future research, namely the Work Productivity and Activity Impairment questionnaire (WPAI), which indicates a decrease in work productivity due to mental or physical health problems. The cross-sectional nature of this research is another important limitation. However, authors controlled for the potential influence of common method variance in this sample.

Future research should test the hypothesized model in other sectors and using a longitudinal design. As our sample size was relatively small, we were not allowed to adopt other more robust statistical procedures such as Structural Equation Modeling–this would require a larger sample due to the complexity and the number of parameters of our research model [53].

Although the present study focuses on physicians, nurses, and hospital staff, these findings could also apply to work environments with a high degree of presenteeism. In fact, participants in this study reported relatively low levels of presenteeism. Therefore, future studies should consider other sectors with high prevalence of presenteeism and also examine whether differences between settings and organizational policies account for all positions or whether they are only focused around certain occupations (i.e., nurses and doctors).

Furthermore, considering that our study only included job resources (autonomy) as antecedents of presenteeism attendance, future studies might also consider other job resources (i.e., compensation, job crafting) or eventually job demands such as role ambiguity, role overload or job insecurity due to the political instability at the country level like in Lebanon.

## Conclusion

The present study provides evidence that professionals from the healthcare sector have a common perception regarding presenteeism climate and that these views are created based on the job demands-resources context. This study extends the organizational attendance research domain by exploring the relationships between jobs resources to presenteeism behaviors. Specifically, it extends previous research [5], [34] by studying how mediators (i.e., presenteeism climate) and moderators (i.e., supervisor support) explain the relationship between job characteristics (i.e., job autonomy) and presenteeism attendance behaviors.

## Supporting information

S1 Dataset (SAV)
